# Reproducibility of patient setup in the seated treatment position: A novel treatment chair design

**DOI:** 10.1002/acm2.12024

**Published:** 2017-01-19

**Authors:** Rachel E. McCarroll, Beth M. Beadle, Danna Fullen, Peter A. Balter, David S. Followill, Francesco C. Stingo, Jinzhong Yang, Laurence E. Court

**Affiliations:** ^1^ Department of Radiation Physics Division of Radiation Oncology The University of Texas MD Anderson Cancer Center Houston TX USA; ^2^ The University of Texas Graduate School of Biomedical Sciences Houston TX USA; ^3^ Department of Radiation Oncology Division of Radiation Oncology The University of Texas MD Anderson Cancer Center Houston TX USA; ^4^ Dipartimento Di Statistica Informatica Applicazioni “G.Parenti” University of Florence Viale Morgagni Florence Italy; ^5^ Department of Imaging Physics Division of Diagnostic Imaging The University of Texas MD Anderson Cancer Center Houston TX USA

**Keywords:** novel treatment positioning, patient positioning, upright treatment

## Abstract

Radiotherapy in a seated position may be indicated for patients who are unable to lie on the treatment couch for the duration of treatment, in scenarios where a seated treatment position provides superior anatomical positioning and dose distributions, or for a low‐cost system designed using a fixed treatment beam and rotating seated patient. In this study, we report a novel treatment chair that was constructed to allow for three‐dimensional imaging and treatment delivery while ensuring robust immobilization, providing reproducibility equivalent to that in the traditional supine position. Five patients undergoing radiation treatment for head‐and‐neck cancers were enrolled and were setup in the chair, with immobilization devices created, and then imaged with orthogonal X‐rays in a scenario that mimicked radiation treatments (without treatment delivery). Six subregions of the acquired images were rigidly registered to evaluate intra‐ and interfraction displacement and chair construction. Displacements under conditions of simulated image guidance were acquired by first registering one subregion; the residual displacement of other subregions was then measured. Additionally, we administered a patient questionnaire to gain patient feedback and assess comparison to the supine position. Average inter‐ and intrafraction displacements of all subregions in the seated position were less than 2 and 3 mm, respectively. When image guidance was simulated, L‐R and A‐P interfraction displacements were reduced by an average of 1 mm, providing setup of comparable quality to supine setups. The enrolled patients, who had no indication for a seated treatment position, reported no preference in the seated or the supine position. The novel chair design provides acceptable inter‐ and intrafraction displacement, with reproducibility equivalent to that reported for patients in the supine position. Patient feedback will be incorporated in the refinement of the chair, facilitating treatment of head‐and‐neck cancer in patients who are unable to lie for the duration of treatment or for use in an economical fixed‐beam setup.

## 1 Introduction

The majority of patients treated with radiation therapy are positioned supine on the treatment couch, with a small proportion prone. The supine treatment position is supported by decades of experience and is suited for the routine practice of three‐dimensional treatment planning with imaging from computed tomography (CT) or magnetic resonance scanners which utilize horizontal bores. However, some patients, particularly those with head‐and‐neck or lung cancers, may develop orthopnea, dyspnea, dysphagia, or other conditions that make lying flat for the duration of treatment difficult or impossible. An upright treatment position can mitigate these difficulties. Additionally, when patients assume a seated or upright position, lung volume and motion are reduced, allowing for sparing of normal tissues and fewer radiation‐induced symptoms.^1^, ^2^, ^3^ Clinicians at our institution have expressed interest in the use of a seated treatment position for patients unable to tolerate standard supine positioning.

In addition to the comfort and dosimetric advantages of treatment in the upright position, this treatment position could allow for the development of a treatment paradigm centered on a fixed treatment beam and seated rotating radiotherapy patient. This delivery approach, still in its infantry, would prove advantageous in the development of a low‐cost linear accelerator system, applicable to low‐ and middle‐income countries. Advantages of this approach in terms of cost, shielding, setup, treatment delivery, machine downtime, and others factors are under investigation.^4^, ^5^, ^6^ Interest from vendors in a fixed‐beam system has further supported this work.

Historically, chairs for radiation therapy have been used primarily as an exception for patients unable to tolerate the lying position and have involved temporary replacement of the treatment couch with an upright unit.^7^, ^8^ Additionally, these previous studies are from an era in which treatment planning was carried out primarily using 2D image acquisition^9^, ^10^, ^11^ and margins which were much more tolerant of positional inaccuracies. The degree of these uncertainties is not well documented in the literature; only one description of an upright system included an assessment of the reproducibility of patient position.^9^

Concerning treatment planning in the upright position, recent studies have explored the feasibility of acquiring cone beam CT scans of seated patients using the onboard imaging capabilities of modern medical linear accelerators by positioning the gantry at 0° degrees, and then rotating the patient couch instead of the gantry.^12^ Studies have also demonstrated the feasibility of using cone beam CT images acquired at the treatment unit for treatment planning.^12^, ^13^, ^14^ This work supports our expectation that we will soon be able to take CBCT images of patients in an upright position for the purpose of treatment planning, by rotating the treatment couch. It has been reported that acquisition of a field of view of 40 cm × 26 cm at isocenter is possible.^12^

Given the above, we have developed a treatment chair suitable for use with standard gantry‐based linear accelerator geometries for head‐and‐neck cancer regions, incorporating measures designed to optimize the reproducibility of inter‐ and intrafraction patient setup. Herein, we report the details of the chair design, inter‐ and intrafraction reproducibility measurements for five head‐and‐neck cancer patients under simulated treatment scenarios, and patient feedback and discuss considerations for future development.

## 2 Methods and materials

### 2.A Chair design

**Figure 1:**
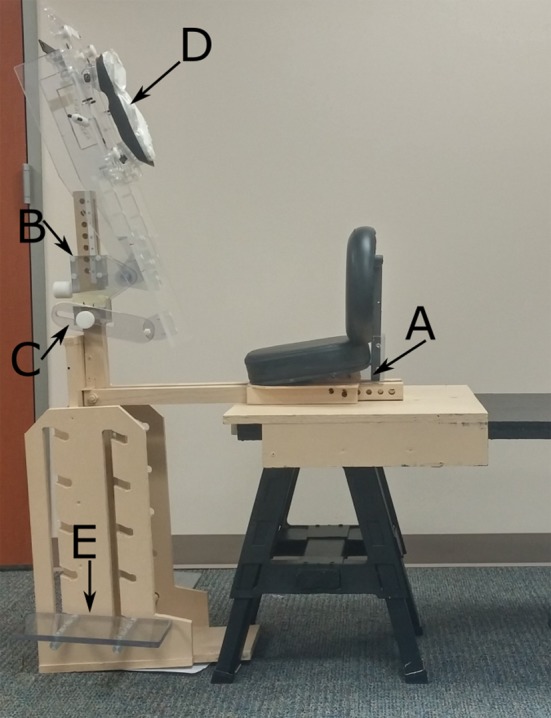
Treatment chair setup. For simulation, a flattop bench was used to mimic the treatment couch in the treatment vault. For image acquisition, the seat was securely fastened to the treatment couch. The setup is adjustable for patient size and comfort including adjustment of the seat depth (a), chest plate height (b), chest plate angle (c), face piece angle (d), and footrest height (e).

The chair was initially designed by engineering students at Rice University (Houston, TX, USA), with major refinement over the last few years to improve patient comfort and ease of patient positioning. The general concept was based on a massage chair, as the forward‐leaning position was expected to give better stability than a regular chair design. Additionally, this forward‐leaning position is beneficial for patients with an excess accumulation of saliva. The chair was constructed in two major parts: (a) the seat with the back rest was constructed such that it slid onto the end of the treatment couch and was securely fastened to avoid shifts in position, and (b) a unit consisting of footrests (15 × 30 × 2 cm acrylic), a chest plate (T‐shaped acrylic), a face piece, and a wooden support post. Once the patient sat down, the second unit slid into position between the patients’ legs and securely tightened into position. Having the chair attach to the couch allowed us to make use of the couch's remote motions to correct patient position based on pretreatment imaging. Additionally, setup of the chair fits smoothly into patient treatment workflow, where therapists gather and position any accessories needed for treatment shortly before the patient enters the treatment vault. The chair allows for many positional variations due to patient size, height, and comfort. Figure 1 shows the available chair adjustments, including adjustment of the seat depth (a), the chest plate height (b), the chest plate angle (c), the face piece angle (d), and the footrest height (e). The chair was manufactured in‐house, primarily from wood and acrylic materials. Limiting the use of metal was important to avoid affecting beam or imaging quality. Furthermore, the construction allowed for easy maneuverability into position. For setup and reproducibility, indexing measures including notches and angle identifiers were incorporated.

### 2.B Intra‐ and interfraction imaging

**Figure 2:**
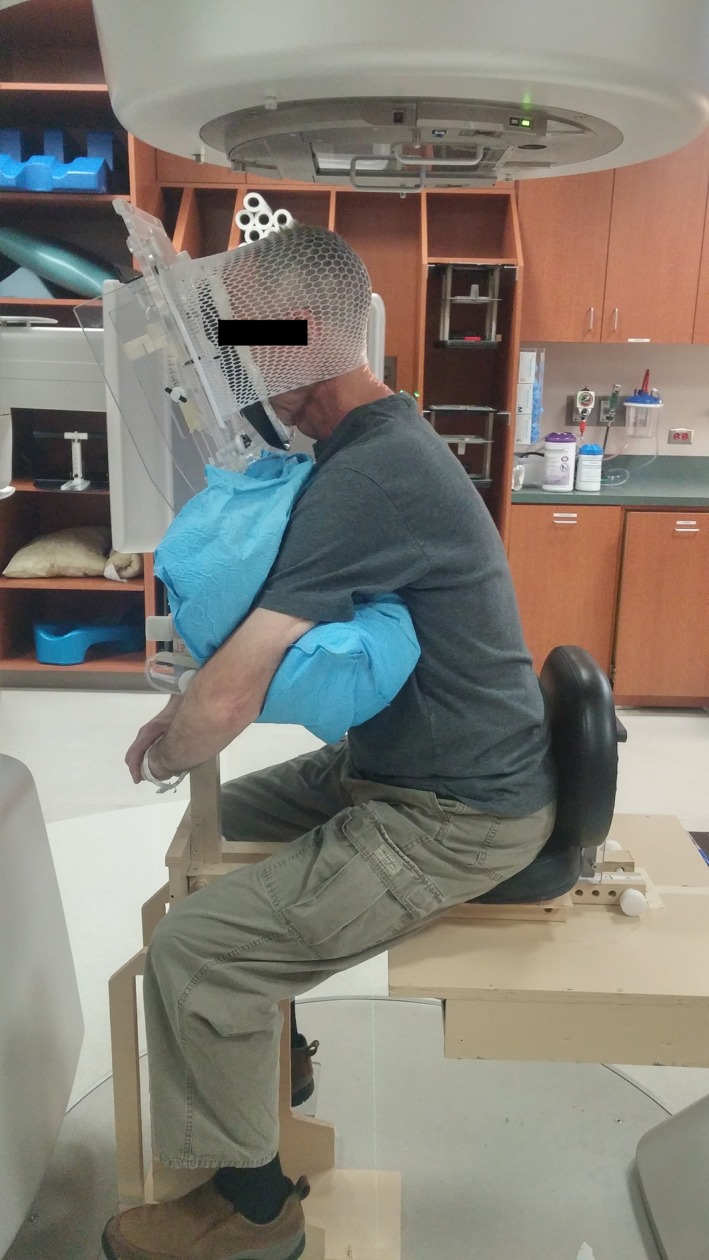
Patient setup for lateral image acquisition in the treatment vault, with kV imagers extended and couch positioned at 0°. The Vac‐Lok cushion was shaped so as to create armrests for patient comfort, the head mask was secured of the back of the patient's head.

Six head‐and‐neck cancer patients undergoing radiotherapy (in a supine position) were accrued with approval from our institutional review board. Five patients completed the study and are included in the analysis. The seated patients were first setup in the treatment chair outside of the treatment room using a flattop bench in lieu of the treatment couch (Fig 1). For setup, the chair position was established and a Vac‐Lok cushion (MTVLG35C; Civco Medical Solutions, Coralville, IA, USA) and thermoplastic head mask (MTAPU; Civco Medical Solutions, Coralville, IA, USA) were made. The Vac‐Lok cushion was used to fill any space between the patient's chest and the chest piece, to create pseudo arm rests for patient comfort, and to facilitate setup reproducibility, especially lateral stabilization (Fig 2). The head mask was secured over the back of the patient's head, in contrast to typical head‐and‐neck cancer treatment for which a thermoplastic mask is generally placed over the patient's face and secured to the treatment table. Additionally, the patients were assessed for need of additional accessories, including an A‐bar for arm and hand positioning and comfort and a pillow behind the back for added support.

For imaging, the chair position was duplicated in the treatment vault. For two patients, acrylic shims were needed to loosen the thermoplastic mask at the face, after the mask had hardened. A TrueBeam^®^ linear accelerator (Varian Medical Systems, Palo Alto, CA, USA) was used for this study, primarily due to the couch end load limit of 200 kg, which allows for positioning of the treatment seat and patient at the couch's end. With the gantry at 330°, kilovoltage imagers retracted, and the patient couch lowered to the full extent and positioned at 270°; the patient was set up as in simulation. The slight gantry rotation was necessary to improve access in this relatively tight space. We also inserted a custom tray into the physical wedge slot to protect the exit window in case of accidental contact.

The longitudinal table position was selected so that the patient's vertebrae were approximately at the beam's isocenter. Using orthogonal lasers, the patient's position was marked on the thermoplastic mask. The gantry was rotated to 0° and kilovoltage imagers were extended outward. The position of kilovoltage imagers varied between patients due to patient size and couch location, to which the chair was attached. The superior–inferior, left–right, and anterior–posterior positions of the imagers relative to the patient ranged 10, 3, and 5 cm, respectively. The position of the imagers was such that anatomical regions captured in the image were similar between patients. Posterior–anterior images were acquired first (couch at 90°, gantry at 0, kV imagers extended), the couch was rotated to 0° and then lateral images were acquired. All mechanical motions occurred under supervision inside the treatment vault.

After image acquisition and under supervision, the couch was rotated for 5 minutes to simulate treatment delivery. Two additional images (lateral and P‐A) were then acquired. Image registration of these two sets of images was used to calculate the first intrafraction reproducibility measurement. The patient then got out of the treatment chair and rested for a few minutes, and the process was repeated to acquire two more sets of images, providing one interfraction reproducibility measurement and one additional intrafraction measurement. Upon completion, the patient was asked to complete a questionnaire (see supplemental materials) regarding both their supine treatments and their experience in the chair.

### 2.C Image registration

**Figure 3:**
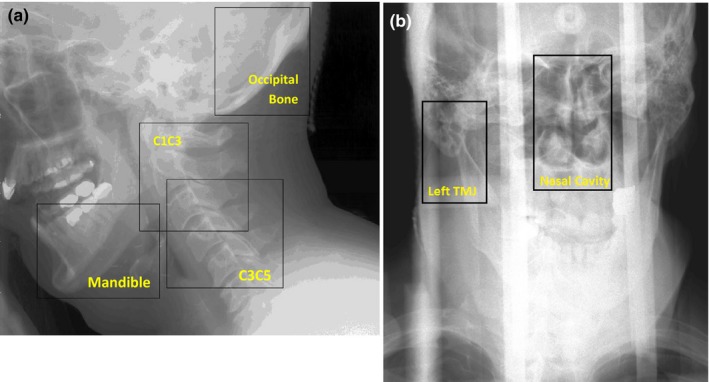
Histogram‐normalized kilovoltage image of a representative patient, outlining the subregions selected on the lateral image (a) and PA image (b) for registration. C1C3 and C3C5, cervical vertebrae 1–3 and 3–5, respectively.

The head‐and‐neck region has many degrees of motion, so inter‐ and intrafraction uncertainties were evaluated for several subregions of the acquired images. We used a method similar to that used previously to evaluate setup uncertainties in patients with head‐and‐neck cancer after cone beam CT guidance.^15^ Subregions of interest on kV projection lateral images were cervical vertebrae 1‐3 (C1C3), C3C5, the mandible, and the occipital bone. The subregions of interest on P‐A images were the left temporomandibular joint and the nasal sinuses. These regions were chosen to facilitate accurate evaluation of patient motion, to match those studied previously,^15^ and to obtain high visibility on the acquired images. The images were processed via histogram normalization and subregions were chosen by hand to include the area of interest, see Fig. 3. Rigid three‐dimensional image registration (bi‐directional translation and rotation) was carried out between the 2 two‐dimensional kilovoltage images in Matlab (MathWorks, Natick, MA, USA) using the gradient descent method with the mean square error as the registration metric. All registration results were verified visually.

### 2.D Simulated image guidance

The use of image guidance for patient positioning and tumor localization in head‐and‐neck cancer patients receiving radiotherapy is a routine procedure in many clinics. We therefore simulated the use of image guidance in our interfraction displacement images, as done by others, which were acquired without patient realignment prior to the simulated treatment delivery. The two images were first registered according to the position of C1C3 (lateral images) or the spinal column (posterior–anterior images). Then, the remaining subregions were registered as previous. This approach provided a measure of the residual error in interfraction displacement given the use of image guidance.

### 2.E Measurement of registration uncertainty

To best approximate the possible uncertainty in the rigid registration, we placed an Alderson Radiation Therapy phantom (ART‐210, Radiology Support Devices, Ramsey, NJ, USA) in the treatment chair, and images were acquired within the range of imaging parameters used to acquire patient images. The chair and phantom were shifted by a known amount and the images were registered. The difference between the registration and the true table position provides a measure of the uncertainty in our rigid registration technique.

## 3 Result and discussion

### 3.A Patients

**Figure 4:**
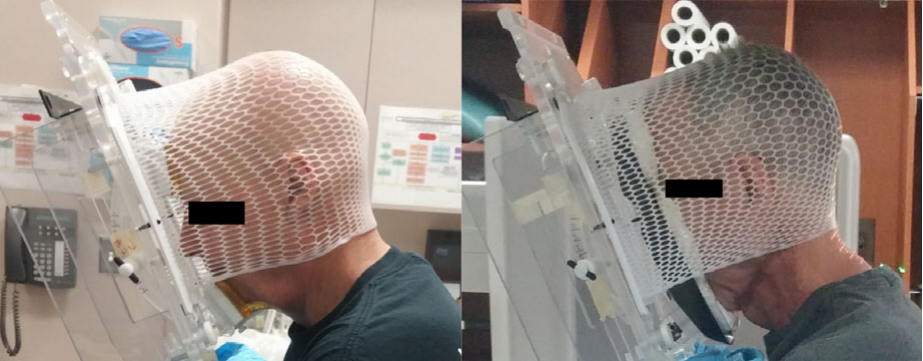
Face piece before and after the change implemented after feedback from the first two patients. Before the change, the chin‐and‐forehead pieces were covered with bolus material for comfort, and the inferior chin piece was arched for anatomical conformity.

The five patients in this study were all male, with a median age of 65 years (range: 55–78 years), mean height of 181.1 cm (range: 180–183 cm), and mean weight of 88 kg (range: 76–111 kg). Female subjects (*n* > 8) were positioned in the chair during trial development and found no difficulties in positioning or comfort. On the basis of the feedback from the first two patients imaged, the face piece was changed from the bolus‐based chin‐and‐forehead piece, to a suctionable cushion which conforms to the patient's face (Fig 4). Furthermore, a small piece of loop fastener was applied to the top of the face piece as a barrier between the seam of the plastic and the patient's forehead.

### 3.B Image registration

Table 1 lists the intra‐ and interfraction displacement for the six subregions measured. Rotation displacement was found to be small, ranging between −0.2° and 0.7°. The error in the registration, as measured with the phantom measurements was found to be no more than 0.4 mm. Average intrafraction displacements were less than 2 mm across all patients. Average interfraction displacements were less than 3 mm. The largest displacements were seen in the anterior–posterior direction. Image guidance improved interfraction patient setup in the anterior–posterior and left–right directions by an average of 1 mm.

**Table 1:** Intrafraction and interfraction displacements with and without image guidance in the treatment chair

Subregion	Mean displacement (mm) ± SE [range] (*n* = 5)								
	Intrafraction			Without IGRT			With IGRT		
	S‐I	A‐P	L‐R	S‐I	A‐P	L‐R	S‐I	A‐P	L‐R
CC13	0.1 ± 1.2 [−1.8 to 1.9]	1.2 ± 2.5 [−3.3 to 4.7]		0.5 ± 2.0 [−1.8 to 3.3]	−2.3 ± 5.3 [−6.8 to 6.1]		Used for IGRT	Used for IGRT	
C3C5	−0.1 ± 1.1 [−1.8 to 1.1]	1.2 ± 3.3 [−4.9 to 5.9]		0.2 ± 2.4 [−3.2 to 3.4]	−2.0 ± 5.7 [−6.8 to 7.1]		−0.3 ± 0.7 [−1.3 to 0.4]	0.3 ± 0.5 [−0.1 to 1.0]	
Mandible	0.1 ± 1.1 [−1.1 to 2.3]	0.5 ± 1.6 [−1.7 to 3.6]		1.0 ± 1.8 [−1.1 to 3.7]	−1.2 ± 4.3 [−7.1 to 4.0]		0.5 ± 1.3 [−1.6 to 2.0]	1.1 ± 3.6 [−2.1 to 7.0]	
Occipital bone	0.2 ± 1.4 [−2.3 to 1.6]	0.3 ± 2.5 [−5.5 to 3.5]		−0.3 ± 2.3 [−3.2 to 3.0]	−2.7 ± 4.3 [−7.4 to 1.5]		−0.8 ± 0.4 [−1.4 to −0.3]	−0.4 ± 2.6 [−4.6 to 2.3]	
Nasal Cavity	0.4 ± 1.8 [−3.2 to 2.4]		−0.7 ± 1.2 [−3.2 to 1.0]	0.4 ± 2.0 [−1.9 to 3.5]		2.1 ± 3.4 [−1.0 to 7.3]	−1.1 ± 0.9 [−1.9 to 0.3]		1.7 ± 6.8 [−9.1 to 7.6]
Left TMJ	0.6 ± 1.6 [−2.5 to 3.0]	−0.8 ± 1.8 [−4.7 to 1.2]		0.3 ± 1.9 [−2.1 to 3.0]		3.0 ± 4.1 [−1.5 to 8.4]	−1.3 ± 1.1 [−2.4 to −0.1]		2.6 ± 4.5 [−4.0 to 7.8]

SE, standard error; S‐I, superior–inferior; A‐P, anterior–posterior; L‐R, left–right; IGRT, image‐guided radiation therapy; C1C3 and C3C5, cervical vertebrae 1–3 and 3–5, respectively; TMJ, temporomandibular joint.

### 3.C Patient questionnaire

**Figure 5:**
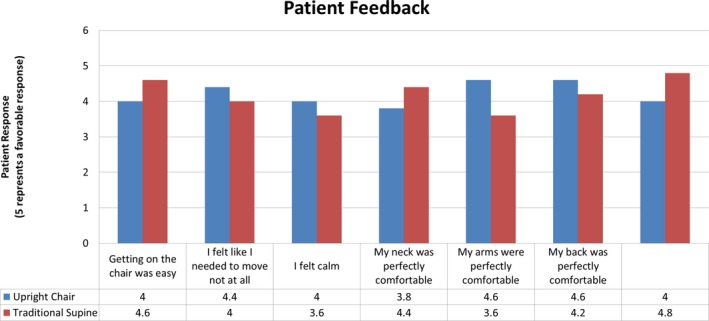
The results of the patient questionnaire. Only questions separated by an average of 0.4 points (5‐point scale) or more are shown. The full questionnaire can be found in the supplemental materials. The questionnaire alternated the score assigned to a positive response. For example, a rating of 5 was assigned to answers of “I felt calm” and “Getting on the chair (couch) was difficult”. In this figure, all positive responses are correlated to ratings of 5, for clarity.

Patients were asked to rate various aspects of their treatment in the supine and seated positions by completing a questionnaire consisting of 15 items. Fourteen of the fifteen resultant comparisons were less than one point apart on a 6‐point (0–5) scale. In Fig. 5, the questions separated by four‐tenths or more of a point are illustrated. Regarding comfort in the arms during treatment, patients preferred the seated position over the supine position, with a mean score of 4.6, compared with 3.6 for the supine position (a score of 5 corresponded to “perfectly comfortable”). Patients also had the opportunity to provide written and verbal feedback about the treatment experience. Feedback included discomfort at the chin and lips, which was partially alleviated with the change in the face piece, as indicated by fewer verbal reports of discomfort after the change was made. Pressure from the head mask and pressure at the chest from the Vac‐Lok cushion were also noted. Several patients expressed the expected benefit of a deeper seat cushion. One patient requested a strap around the back to help prevent slouching and to remind the patient to relax forward into the chair.

### 3.D Discussion

As radiation therapy treatment planning has moved almost entirely to three‐dimensional methods, the acquisition of CT scans for planning has become routine in many clinics around the world. The horizontal bore of such scanners is a limiting factor for possible patient positions. Nearly all patients are therefore treated lying in a prone or supine position. However, this position may not be suitable for all patients, especially those suffering from orthopnea, dyspnea, or dysphagia. Furthermore, dosimetric considerations may indicate upright or seated patient positioning. As techniques for image acquisition at the treatment unit continue to advance, for treatment paradigms still reliant on two‐dimensional planning techniques, for patients unable to tolerate a lying treatment position, and for the development of a fixed‐beam low‐cost system an upright treatment chair may prove optimal for treatment. We have designed a treatment chair compatible with current linear accelerator geometries and have tested patient intra‐ and interfraction displacement for the head‐and‐neck region. Patient displacement was on average less than 2 mm in the intra‐ and 3 mm in the interfraction scenarios. These raw interfraction measurements prove much better than those found for a previous upright setup for mantle treatments, for which all patients required block shifts of at least 5 mm, and 35% requiring shifts greater than or equal to 1 cm.^9^

We also evaluated interfraction displacements in a scenario of simulated image guidance. While in clinical scenarios, the radiation therapist would typically compare the whole acquired image to a planning image for use in image guidance, we have used only a subregion of the acquired image to simulate image guidance. This approach is consistent with techniques used previously,^15^ and both the mean and standard deviation of interfraction displacement in the seated position in our study are on the same order as those reported for the traditional supine position techniques (Table 2).

**Table 2:** Comparison of interfraction displacements in the seated and supine treatment positions for simulated image guidance with respect to cervical vertebrae 1–3

Region of interest	Mean displacement (mm) ± SE		
	Upright position (this study)	Supine position	
		Kapanen et al.^16^, *	van Kranen et al.^15^
Cranial–caudal			
C3C5	−0.3 ± 0.7	1.2*	0.10 ± 0.00
Mandible	0.5 ± 1.3	2.9	1.30 ± 2.50
Occipital bone	−0.8 ± 0.4	1.3	0.60 ± 2.0 0
Anterior–posterior			
C3C5	0.3 ± 0.5	3.1*	0.10 ± 0.50
Mandible	−1.1 ± 3.6	2.2	−0.30 ± 1.20
Occipital bone	−0.4 ± 2.6	1.9	0.30 ± 0.60

*Standard errors (SE) were not reported by Kapanen et al.

Additionally, cervical vertebrae 1–2 (C1C2) were used as a reference, and C5C7 data were reported instead of C3C5 data.

There are limitations to this technique. One patient was not able to complete the testing, and review of his images before the trial was aborted suggests that he had significant intrafractional displacement (up to 3.3 cm). This was likely due to the fact that he was falling asleep and not feeling well, resulting in significant positional changes. While this only affected one patient, this may be more widespread; our attempts to create a treatment chair that is better tolerated than the supine position may not be tolerated by some patients. Furthermore, we largely enrolled “healthy” patients who tolerated the supine position quite well, and they also tolerated the upright position quite well. It remains to be seen how patients with significant medical issues (for instance, orthopnea, dyspnea, thick secretions) tolerate the upright treatment, and whether it reflects an improvement over the supine. These issues will be investigated in future studies.

The tight geometry of the gantry system and the chair tested in our study is partially a result of a minimum vertical height of the treatment couch and seat height; therefore, care must be taken when positioning the patient. We estimate that by the sixth patient, setup took approximately 8 minutes including marking of lasers on head mask, similar to that for supine positioning.

A complete assessment of the shift accuracy of the used registration algorithm was completed. However, while the rotational displacement of patient images was small, less than 1°, a similar analysis was not completed for the rotational accuracy of the registration algorithm and is potential source of error in this study.

The ability to acquire treatment planning images in the upright position, mirroring that of treatment, is an important aspect of the complete treatment process in the upright position. Using onboard imaging systems or other techniques, the acquisition of planning images in the treatment position would get around issues of organ deformation faced by others. When considering other treatment sites, abdominal and pelvic cavities, especially, considerations of organ deformation (day to day, etc.) will need special attention. Herein we have explored the setup reproducibility of the upright treatment position in an in‐house built chair.

We are currently in the process of modifying the chair design to reflect patient feedback and our accumulated experience. Projected changes include the removal of the stand, this will require more sturdy construction and counterbalancing, a lower seat which will relieve part of the tightness of the geometry, and the incorporation of a small pillow or strap into the patient seat to facilitate a feeling of stability and safety in the forward‐leaning position. Furthermore, to allow manipulation of the chair position to fit many patients, full indexing of such motions will be incorporated. Additionally, dosimetric considerations given the observed inter‐ and intrafraction displacement will be considered.

In conclusion, our preliminary tests indicate that it is feasible to create an upright treatment chair with geometry suitable for 3‐D imaging (with cone beam CT) and robust reproducible patient position between and within radiotherapy fractions. Our findings, in conjunction with feedback from the patients’ questionnaires, will guide adaptation of the current chair design. Our goal was to create a system whereby patients can be simulated and treated in an upright position without degradation of a conformal, modern radiation treatment plan.

## Conflict of Interest

This work was supported in part by Varian Medical Systems.
